# Use of MenACWY-CRM Vaccine in Children Aged 2 Through 23 Months at Increased Risk for Meningococcal Disease: Recommendations of the Advisory Committee on Immunization Practices, 2013

**Published:** 2014-06-20

**Authors:** Jessica R. MacNeil, Lorry Rubin, Lucy McNamara, Elizabeth C. Briere, Thomas A. Clark, Amanda C. Cohn

**Affiliations:** 1 Meningitis and Vaccine Preventable Diseases Branch, Division of Bacterial Diseases, National Center for Immunization and Respiratory Diseases, CDC; 2 Advisory Committee on Immunization Practices Meningococcal Vaccines Work Group; 3 EIS officer, CDC

During its October 2013 meeting, the Advisory Committee on Immunization Practices (ACIP) recommended use of a third meningococcal conjugate vaccine, MenACWY-CRM (Menveo, Novartis), as an additional option for vaccinating infants aged 2 through 23 months at increased risk for meningococcal disease. MenACWY-CRM is the first quadrivalent meningococcal conjugate vaccine licensed for use in children aged 2 through 8 months. MenACWY-D (Menactra, Sanofi Pasteur) is recommended for use in children aged 9 through 23 months who are at increased risk for meningococcal disease (1), and Hib-MenCY-TT (MenHibrix, GlaxoSmithKline) is recommended for use in children aged 6 weeks through 18 months at increased risk (Table) (2). This report summarizes information on MenACWY-CRM administration in infants and provides recommendations for vaccine use in infants aged 2 through 23 months who are at increased risk for meningococcal disease (3). Because the burden of meningococcal disease in infants is low in the United States and the majority of cases that do occur are caused by serogroup B, which is not included in any vaccine licensed in the United States, only those infants who are at increased risk for meningococcal disease are recommended to receive a meningococcal vaccine.

*Recommendations for routine use of vaccines in children, adolescents and adults are developed by the Advisory Committee on Immunization Practices (ACIP). ACIP is chartered as a federal advisory committee to provide expert external advice and guidance to the Director of the Centers for Disease Control and Prevention (CDC) on use of vaccines and related agents for the control of vaccine-preventable diseases in the civilian population of the United States. Recommendations for routine use of vaccines in children and adolescents are harmonized to the greatest extent possible with recommendations made by the American Academy of Pediatrics (AAP), the American Academy of Family Physicians (AAFP), and the American College of Obstetrics and Gynecology (ACOG). Recommendations for routine use of vaccines in adults are harmonized with recommendations of AAFP, ACOG, and the American College of Physicians (ACP). ACIP recommendations adopted by the CDC Director become agency guidelines on the date published in the* Morbidity and Mortality Weekly Report (MMWR)*. Additional information regarding ACIP is available at http://www.cdc.gov/vaccines/acip.*

## Methods

In monthly teleconferences, the ACIP Meningococcal Vaccines Work Group reviewed safety and immunogenicity data from five phase 3 clinical trials of MenACWY-CRM use in infants aged 2 through 23 months (4–8). Data on the concomitant administration of MenACWY-CRM and 7-valent pneumococcal conjugate vaccine (PCV7) were discussed by both the Meningococcal and Pneumococcal ACIP work groups. The Meningococcal Vaccines Work Group also reviewed published peer-reviewed literature and unpublished data on disease epidemiology. Evidence of benefits, harms, values and preferences, and cost-effectiveness were reviewed in accordance with GRADE methods (9). A summary of the data reviewed and work group discussions were presented to ACIP, and recommendations for use of MenACWY-CRM in infants were approved by ACIP at its October 23–24, 2013 meeting.

## Summary of Data Reviewed

MenACWY-CRM is a conjugate vaccine in which the capsular polysaccharides from *Neisseria meningitidis* serogroups A, C, W, and Y are conjugated to the diphtheria toxin mutant CRM197 (10). The vaccine provides protection against meningococcal serogroups A, C, W, and Y, but not against serogroup B.

Immunogenicity of MenACWY-CRM in infants aged 2 through 23 months was evaluated in four clinical trials (4–7). In all trials, enrolled subjects were randomized to receive either routine infant vaccinations and MenACWY-CRM, or routine infant vaccines alone. Human serum bactericidal antibody (hSBA) titers were used as a correlate of protection to assess vaccine immunogenicity; hSBA titers ≥1:8 were considered protective (4,7). Two open-label, multicenter trials assessed immunogenicity of a 4-dose MenACWY-CRM series, with doses at ages 2, 4, 6, and 12 months (4,7). A third trial assessed immunogenicity of a 4-dose series, with doses at ages 2, 4, 6, and 16 months, and a 3-dose series, with doses at ages 2, 6, and 12 months (6). A fourth randomized, open-label, multicenter trial assessed immunogenicity of a 2-dose MenACWY-CRM regimen, with doses at ages 7 through 9 months and 12 months (5). In the first three trials, hSBA titers were assessed 1 month after completion of the infant series (age 7 months) and 1 month after completion of the full series (age 13 or 17 months, depending on the dose regimen used) (4,6,7). In the fourth trial, hSBA titers were assessed 1 month after completion of the 2-dose series, at age 13 months.

In the first three trials, 1 month after completion of a 3-dose infant series, 67%–89% of subjects had protective hSBA titers for serogroup A, and 94%–98% had protective hSBA titers for serogroups C, W, and Y (4,6,7). In the first two trials, at age 13 months, 89%–94% of infants achieved protective hSBA antibody titers for serogroup A, and 95%–100% achieved protective hSBA antibody titers for the remaining serogroups (4,7); similar immune responses were observed at ages 13 and 17 months, respectively, among infants who received a 2-dose series (age 7 through 9 and 12 months) in the fourth trial or a 3-dose infant series with a 16-month toddler dose (5,6,11) in the third trial. Among infants receiving doses at ages 2, 6, and 12 months, 74% had protective hSBA titers for serogroup A, and at least 94% had protective hSBA titers for serogroups C, W, and Y at age 7 months; at least 94% had protective hSBA titers against all four serogroups at age 13 months (6). Preliminary results suggest that 2 years after a 4-dose MenACWY-CRM series (at ages 2, 4, 6, and 12 months) is completed, 34%–76% of children maintain protective hSBA titers for serogroups C, Y, and/or W (Novartis, unpublished data, 2013). However, protection against serogroup A wanes in almost all children by this time (Novartis, unpublished data, 2013).

Three trials evaluated concomitant administration of MenACWY-CRM and routine childhood vaccinations. Interference with immune responses to pneumococcal serotypes 6B (4,7) and 23F (4) was suggested after PCV7 coadministration with the 3-dose infant series of MenACWY-CRM compared with infants receiving PCV7 alone in two trials (4,7,11). However, after concomitant administration of PCV7 and the 12-month dose of MenACWY-CRM, no reduction in responses to pneumococcal serotype 6B or 23F was observed (4,7,11).

No interference with the immune response was observed for pertussis antigens based on geometric mean concentration ratios (4–7). Seroresponses to other pneumococcal serotypes and to antigens in other routine childhood vaccines (diphtheria-tetanus-acellular pertussis-inactivated poliovirus-*Haemophilus influenzae* type b combined vaccine [DTaP-IPV-Hib] [Pentacel, Sanofi Pasteur]); hepatitis B virus vaccine; measles, mumps, and rubella vaccine; and measles, mumps, rubella, and varicella vaccine were not affected by MenACWY-CRM administration (4,5,11).

The work group also examined data from the five clinical trials evaluating adverse events in infants receiving MenACWY-CRM (4–8,11). Among approximately 5,000 subjects studied through 6-months postvaccination, local and systemic adverse events after administration of MenACWY-CRM and routine vaccinations were similar to those observed after routine vaccination alone (4–8,11); 11 serious adverse events were considered possibly related to MenACWY-CRM.* No deaths were considered related to MenACWY-CRM.

## Recommendations

Vaccination with an age- and formulation-appropriate meningococcal conjugate vaccine is recommended for infants aged 2 through 23 months at increased risk for meningococcal disease. As described previously (1–3), infants at increased risk for meningococcal disease are:

- those with persistent complement component deficiencies (C3, C5–C9, properdin, factor D, and factor H),
- those with functional or anatomic asplenia (including sickle cell disease),
- healthy infants in communities with a meningococcal disease outbreak for which vaccination is recommended, and
- those traveling to or residing in areas where meningococcal disease is hyperendemic or epidemic.

Routine vaccination against meningococcal disease is not recommended for children aged 2 months through 10 years.

MenACWY-CRM may be used for protection against serogroups A, C, W, and Y in infants aged 2 through 23 months who are recommended for meningococcal vaccination because of an increased risk for meningococcal disease (Table). Infants are recommended to receive a 4-dose vaccination series, with doses at ages 2, 4, 6, and 12 months. Children initiating vaccination at ages 7 through 23 months are recommended to receive 2 doses of MenACWY-CRM, with the second dose administered at age ≥12 months and ≥3 months after the first dose. MenACWY-CRM is the only vaccine licensed for infants aged <9 months that includes protection against meningococcal serogroups A and W; therefore, infants aged <9 months traveling to or residing in areas with hyperendemic or epidemic meningococcal disease caused by these serogroups should receive MenACWY-CRM before travel. Hib-MenCY-TT does not provide protection against serogroups A and W and should not be used for protection in infants traveling to or residing in areas with hyperendemic or epidemic meningococcal disease. Recommendations for use of the other infant meningococcal vaccines, MenACWY-D and Hib-MenCY-TT, have been published previously and remain unchanged (Table) (1–3).

Because of differences in serogroup composition and licensure indication, the same vaccine product should be used for all doses in infants at increased risk for meningococcal disease. However, if the product used for prior doses is unknown or unavailable, the vaccination series can be completed with any age- and formulation-appropriate meningococcal vaccine. Although no data are available on interchangeability of meningococcal vaccines in infants, limited data from a postlicensure study in adolescents suggests safety and immunogenicity of MenACWY-CRM are not adversely affected by prior immunization with MenACWY-D (12).

In previous recommendations, children with functional or anatomic asplenia (including sickle cell disease) were recommended to receive 13-valent pneumococcal conjugate vaccine (PCV13) according to the normal schedule but to delay MenACWY-D vaccination until age 2 years because of immune interference (1,3). Because MenACWY-CRM does not demonstrate immune interference with PCV7 after the 12-month dose, it can be administered concomitantly with PCV13. ACIP recommends that infants aged 2 through 23 months with functional or anatomic asplenia either receive MenACWY-CRM or Hib-MenCY-TT or wait until age 2 years to receive MenACWY-D (Table).

For children at prolonged increased risk for meningococcal disease, ACIP recommends booster doses of conjugate meningococcal vaccine after completion of the primary series. As stated previously (1), if the most recent dose was received before age 7 years, a booster dose should be administered 3 years later. Additional boosters should be administered every 5 years thereafter.

## Figures and Tables

**TABLE:** Summary of recommendations for meningococcal vaccination of children aged 2–23 months at increased risk for meningococcal disease — Advisory Committee on Immunization Practices, 2013

Vaccine	Age of primary vaccination	Booster doses*	Indicated for infants who:	Not indicated for:
MenACWY-CRM (Menveo)	2, 4, 6, and 12 months	1st booster 3 years after primary series Additional boosters every 5 years	Have complement component deficiencies Have functional or anatomic asplenia (including sickle cell disease) Are in the risk group for an outbreak for which vaccination is recommended Are traveling to or residing in regions where meningitis is epidemic or hyperendemic	
MenACWY-D (Menactra)	9 and 12 months†	1st booster 3 years after primary series Additional boosters every 5 years	Have complement component deficiencies Are in the risk group for an outbreak for which vaccination is recommended Are traveling to or residing in regions where meningitis is epidemic or hyperendemic	Infants with functional or anatomic asplenia (including sickle cell disease)§
Hib-MenCY-TT (MenHibrix)	2, 4, 6, and 12–15 months	1st booster (using MenACWY-CRM or MenACWY-D¶) 3 years after primary series	Have complement component deficiencies	Infants traveling internationally to regions where meningitis is epidemic or hyperendemic
		Additional boosters (using MenACWY-CRM or MenACWY-D¶) every 5 years	Have functional or anatomic asplenia (including sickle cell disease) Are in the risk group for an outbreak for which vaccination is recommended	Booster dose in children aged >18 months

* If the most recent dose was received before age 7 years, a booster dose should be administered 3 years later.

† For infants aged 9–23 months, 2 doses of MenACWY-D should be administered 12 weeks apart. For infants receiving the vaccine before travel, the second dose may be administered as soon as 8 weeks after the first dose (additional information at http://www.cdc.gov/mmwr/preview/mmwrhtml/rr6202a1.htm).

§ Because of high risk for invasive pneumococcal disease, children with functional or anatomic asplenia should not be immunized with MenACWY-D before age 2 years to prevent immune interference with 13-valent pneumococcal conjugate vaccine (PCV13).

¶ Hib-MenCY-TT should not be used for booster doses. A quadrivalent meningococcal vaccine (MenACWY-CRM or MenACWY-D) should be used for booster doses.

## Footnotes

* The administration of the investigational vaccine and an adverse event were considered reasonably related in time and the adverse event could be explained by causes other than exposure to the investigational vaccine. Reported adverse events included acute encephalomyelitis (one case), cellulitis (one), complex partial seizure (one), epilepsy (one), febrile seizure (three), fever (one), Kawasaki disease (three).
